# Highly Pathogenic Avian Influenza A(H5N1) Virus Clade 2.3.4.4b Infections in Wild Terrestrial Mammals, United States, 2022

**DOI:** 10.3201/eid2912.230464

**Published:** 2023-12

**Authors:** Elizabeth J. Elsmo, Arno Wünschmann, Kimberlee B. Beckmen, Liam E. Broughton-Neiswanger, Elizabeth L. Buckles, Jayne Ellis, Scott D. Fitzgerald, Robert Gerlach, Shawna Hawkins, Hon S. Ip, Julia S. Lankton, Erin M. Lemley, Julianna B. Lenoch, Mary L. Killian, Kristina Lantz, Lindsey Long, Roger Maes, Marta Mainenti, Julie Melotti, Megan E. Moriarty, Shotaro Nakagun, Rachel M. Ruden, Valerie Shearn-Bochsler, Danielle Thompson, Mia K. Torchetti, Arnaud J. Van Wettere, Annabel G. Wise, Ailam L. Lim

**Affiliations:** University of Wisconsin–Madison School of Veterinary Medicine, Madison, Wisconsin, USA (E.J. Elsmo, S. Hawkins, E.M. Lemley, A.L. Lim);; Wisconsin Veterinary Diagnostic Laboratory, Madison (E.J. Elsmo, A.L. Lim);; Minnesota Veterinary Diagnostic Laboratory, St. Paul, Minnesota, USA (A. Wünschmann);; Alaska Department of Fish and Game, Fairbanks, Alaska, USA (K.B. Beckmen);; Washington Animal Disease Diagnostic Laboratory, Pullman, Washington, USA (L.E. Broughton-Neiswanger);; New York State Animal Health Diagnostic Center, Ithaca, New York, USA (E.L. Buckles, S. Nakagun);; Michigan State University Veterinary Diagnostic Laboratory, Lansing, Michigan, USA (J. Ellis, S.D. Fitzgerald, R. Maes, D. Thompson, A.G. Wise);; Alaska Department of Environmental Conservation, Anchorage, Alaska, USA (R. Gerlach);; National Wildlife Health Center, Madison (H.S. Ip, J.S. Lankton, V. Shearn-Bochsler);; Dane County Humane Society’s Wildlife Center, Madison (E.M. Lemley);; US Department of Agriculture National Wildlife Research Center, Fort Collins, Colorado, USA (J.B. Lenoch);; US Department of Agriculture Animal and Plant Health Inspection Service, Ames, Iowa, USA (M.L. Killian, K. Lantz, M.K. Torchetti);; Wisconsin Department of Natural Resources, Madison (L. Long);; Iowa State University Veterinary Diagnostic Laboratory, Ames (M.E. Mainenti, R.M. Ruden);; Michigan Department of Natural Resources, Lansing (J. Melotti, M.E. Moriarty);; Iowa Department of Natural Resources, Ames (R.M. Ruden);; Utah Veterinary Diagnostic Laboratory, Logan, Utah, USA (A.J. Van Wettere)

**Keywords:** influenza, highly pathogenic avian influenza virus, avian influenza, influenza A(H5N1), viruses, clade 2.3.4.4b, respiratory infections, wild terrestrial mammals, fox, bobcat, raccoon, skunk, opossum, coyote, fisher, meningitis/encephalitis, zoonoses, United States

## Abstract

We describe the pathology of natural infection with highly pathogenic avian influenza A(H5N1) virus of Eurasian lineage Goose/Guangdong clade 2.3.4.4b in 67 wild terrestrial mammals throughout the United States during April 1‒July 21, 2022. Affected mammals include 50 red foxes (*Vulpes vulpes*), 6 striped skunks (*Mephitis mephitis*), 4 raccoons (*Procyon lotor*), 2 bobcats (*Lynx rufus*), 2 Virginia opossums (*Didelphis virginiana*), 1 coyote (*Canis latrans*), 1 fisher (*Pekania pennanti*), and 1 gray fox (*Urocyon cinereoargenteus*). Infected mammals showed primarily neurologic signs. Necrotizing meningoencephalitis, interstitial pneumonia, and myocardial necrosis were the most common lesions; however, species variations in lesion distribution were observed. Genotype analysis of sequences from 48 animals indicates that these cases represent spillover infections from wild birds.

Since October 2021, outbreaks of highly pathogenic avian influenza (HPAI) A(H5N1) virus belonging to A/Goose/Guangdong/1/1996 lineage H5 clade 2.3.4.4b have been reported throughout Europe (*1*). Transatlantic spread of HPAI H5N1 virus with genetic similarity to Eurasian lineages was detected in the United States in December 2021 and has subsequently spread throughout the continental United States in wild birds and domestic poultry (*2**‒**6*).

In addition to disease outbreaks in domestic poultry, currently circulating HPAI H5N1 virus is persisting in wild bird reservoirs, with multiple reports of spillover into and clinical infection in various mammal species in countries in Europe during 2021 (*1*,*3**,**7*). We report a case series on the pathology of natural infections with HPAI H5N1 virus in terrestrial wild mammals in the United States concurrent with high levels of circulating HPAI viruses in wild birds during the spring and summer of 2022.

## Materials and Methods

Case inclusion criteria were confirmed positivity for HPAI H5N1 virus infection by real-time reverse transcription PCR (rRT-PCR) or, in 3 cases, by having consistent lesions, positive avian influenza virus immunohistochemistry (IHC) results, and being part of a litter with other animals confirmed positive by rRT-PCR. The cases represent opportunistic samples from wild mammals reported ill or dead by citizens and submitted to state wildlife agencies or veterinary diagnostic laboratories for diagnostic purposes. Clinical observations were recorded by citizens, wildlife professionals, rehabilitators, or veterinary professionals. Except for 2 red foxes, for which only antemortem samples were available, all animals had postmortem examinations performed by veterinarians or pathologists. Postmortem samples included swab specimens in viral transport medium, tissue samples stored refrigerated or frozen, and tissues fixed in 10% neutral-buffered formalin.

We used the standardized protocols for the National Animal Health Laboratory Network for HPAI virus rRT-PCR testing on a variety of sample types. We extracted total nucleic acids from samples by using the KingFisher Flex or KingFisher Purification System platforms and MagMAX-96 Viral RNA Isolation Kit or MagMAX Pathogen RNA/DNA Kit (Life Technologies, https://www.thermofisher.com) according to the manufacturer’s protocol. We also performed a general influenza A virus (IAV) rRT-PCR targeting the conserved region of the IAV matrix gene (*8*,*9*). We performed further subtyping by using 2 IAV H5 subtyping assays: the avian influenza H5 subtype rRT-PCR targeting the hemagglutinin gene for the North American, Eurasian, and Mexican lineage of avian influenza (*8**–**11*) and an H5 2.3.4.4b-specific rRT-PCR developed in collaboration with the US Department of Agriculture Southeast Research Laboratory (SEPRL; Real-Time RT-PCR Assay for the Detection of Goose/Guangdong lineage Influenza A subtype H5, clade 2.3.4.4; NVSL-WI-1732).

Influenza A–positive samples were sent to the National Veterinary Services Laboratories (NVSL) in Ames, Iowa for confirmation and characterization. Testing at NVSL included an H5 clade 2.3.4.4 pathotyping assay and an assay targeting neuraminidase 1 (N1) for rapid pathotyping (SEPRL; Real-Time RT-PCR Assay for Pathotyping Goose/Guangdong lineage Influenza A subtype H5, clade 2.3.4.4; NVSL-WI-1767) and neuraminidase subtyping (SEPRL; Real-Time RT-PCR Assay for the Detection of Eurasian-lineage Influenza A Subtype N1; NVSL-WI-1768). Influenza A viruses were sequenced directly as previously described (*4*). We used RAxML (https://github.com/amkozlov/raxml-ng) to generate phylogenetic trees and created tables of single-nucleotide polymorphisms (SNPs) by using the vSNP pipeline (https://github.com/USDA-VS/vSNP) with a reference composed of 6 segments from an H5N1 2.3.4.4b clade virus and 2 segments from North America–origin wild bird viruses (Appendix Table 1). Routine detection of mutations associated with mammalian adaptation at the consensus level uses the designated SNP functionality of the vSNP pipeline, generating an SNP table of all sequences with mutations at the assigned position. The included locations are in the polymerase basic 2 (PB2) gene: position 1894 for E627K, position 2116 for D701N, and position 826 for T271A. We used a search of the variant call files generated by the vSNP pipeline to generate a list of both consensus and subconsensus level presence of the designated variants. Genotypes were assigned according to the scheme described in Youk et al. (*3*), using designated SNPs in the vSNP pipeline, as well as the GenoFLU tool (https://github.com/USDA-VS/GenoFLU).

In some cases, additional ancillary testing was performed. Formalin-fixed tissues were processed for histopathologic analysis and evaluated by veterinary pathologists at multiple institutions. A subset of tissues from some animals was processed for monoclonal IHC analysis for IAV, canine distemper virus antigen, or both. All ancillary testing procedures were performed according to validated procedures at federal and state diagnostic laboratories accredited by the American Association of Veterinary Laboratory Diagnosticians.

## Results

**Figure 1:**
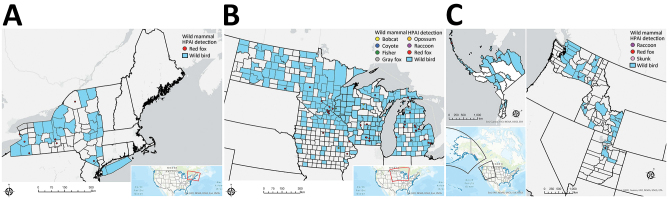
Locations of wild mammals naturally infected with HPAI A(H5N1) virus, United States, April‒June 2022. A) Eastern region; B) midwestern region; C) western region. If exact location could not be identified, the county centroid was used. Blue indicates counties with positive detections of HPAI H5N1 in wild birds during March‒August 2022. HPAI, highly pathogenic avian influenza.

During April 1‒July 21, 2022, HPAI virus was detected in 67 wild mammals from 10 states: Alaska, Idaho, Iowa, Michigan, Minnesota, New York, North Dakota, Utah, Washington, and Wisconsin (Figure 1). Species identified were 50 red foxes (*Vulpes vulpes*), 6 striped skunks (*Mephitis mephitis*), 4 raccoons (*Procyon lotor*), 2 bobcats (*Lynx rufus*), 2 Virginia opossums (*Didelphis virginiana*), 1 coyote (*Canis latrans*), 1 fisher (*Pekania pennanti*), and 1 gray fox (*Urocyon cinereoargenteus*) (Appendix Table 2).

Based on weight and dentition, all red foxes, striped skunks, opossums, 3 of 4 raccoons, and the coyote were juveniles, and 1 raccoon, both bobcats, the fisher, and the gray fox were adults (*12*). Within species with >4 animals, no sex predilections were apparent (Appendix Table 2). Six pairs of red foxes and 2 opossums were known or strongly suspected to be littermates, and skunks 1‒5 comprised 2 litters that were comingled at a rehabilitation center.

Nine animals were found dead. Of those found alive, 13 were euthanized in the field and 44 were under the care of a wildlife rehabilitator or veterinarian (Appendix Table 2). Of those 44 animals, 2 red foxes remained alive and were clinically normal before release, 13 died, and the remainder were euthanized. The outcome was not reported for 1 animal.

Among the 58 animals found alive, most (n = 54) had neurologic abnormalities, comprising seizures (n = 29), ataxia (n = 23), tremors (n = 17), lack of fear of humans (n = 7), vocalization (n = 5), circling (n = 4), blindness (n = 3), torticollis (n = 2), nystagmus (n = 1), and grimace (n =1) (Appendix Table 3). Less frequently recorded abnormalities were lethargy (n = 28), fever (n = 7), diarrhea (n = 2), unconsciousness (n = 2), recumbence (n = 1), paralysis (n = 1), and vomiting (n = 1). Dyspnea was reported in 2 skunks, 1 bobcat, and 1 red fox.

**Figure 2,:**
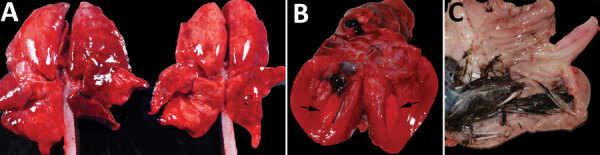
Gross photographs of postmortem lesions from red foxes naturally infected with highly pathogenic avian influenza virus, United States. A) Lungs have failed to collapse and are diffusely edematous and mottled pink to dark red. B) Cross section of the left ventricle of the heart showing a focal region of myocardial pallor in the papillary muscle (arrows). C) Stomach contents with feathers.

Gross postmortem observations were recorded in 58 animals. Most animals were in fair to good nutritional condition (n = 39). Lung lesions were consistently observed (n = 49) and included congestion (n = 42), edema (n = 22), failure to collapse (n = 18), hemorrhage (n = 18), and pleural effusion (n = 6) (Figure 2, panel A). The most common brain lesions were hemorrhage (n = 11) and congestion (n = 7). Other lesions included pallor (n = 8), congestion (n = 7), enlargement (n = 6), and hemorrhage (n = 1) in the liver, as well as congestion (n = 4) and cortical hemorrhage (n = 1) in the kidney. Pericardial effusion (n = 3), petechia (n = 2), and myocardial pallor (n = 2) were infrequently noted in the heart (Figure 2, panel B). In the gastrointestinal tract, nematode parasitism was relatively common (n = 15), but other rarely observed lesions included congestion, hemorrhage, and loose feces. Three red foxes had gastric contents that included feathers (Figure 2, panel C). Three red foxes had mild ocular discharge. The gray fox had severe hemorrhage in all body cavities.

**Figure 3:**
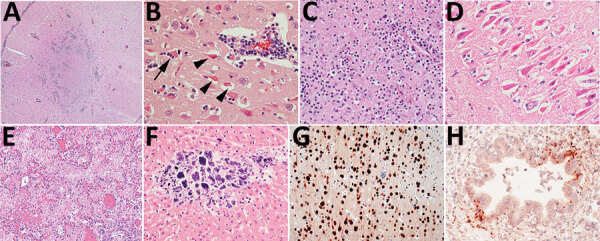
Histopathology of lesions in red foxes naturally infected with highly pathogenic avian influenza virus, United States. A) Throughout the brain, there are multifocal regions of necrosis and hypercellularity. Original magnification ×4. B) Within the gray matter, there is prominent neuronal necrosis (arrowheads), satellitosis (arrow), and reactive astrocytes. A vessel is surrounded by lymphocytes and plasma cells. Original magnification ×4. C) In areas of necrosis within the brain, there is often abundant, stippled, basophilic karyorrhectic debris. Original magnification ×40. D) Within the hippocampus, there are numerous shrunken, angular, and acidophilic (necrotic) neurons in a laminar pattern. Original magnification ×20. E) Within the lung, there is diffuse vascular congestion. Alveoli contain fibrin, hemorrhage, and edema fluid. Original magnification ×20. F) Regions of cardiomyocyte necrosis in the heart are often mineralized. Original magnification ×40. Panels A‒F, hematoxylin and eosin stain.G) Within the brain, there is positive nuclear and cytoplasmic staining of neuron cell bodies and processes. Avian influenza virus monoclonal immunohistochemical analysis. Original magnification ×40. H) Scattered positive nuclear and cytoplasmic staining of bronchiolar epithelial cells and interstitial macrophages in the lung. Monoclonal immunohistochemical analysis of influenza A virus nucleoprotein. Original magnification ×40.

Histopathologic findings were recorded for 55 animals (Table 1; Appendix Table 4). Of 54 animals with >1 section of brain suitable for histopathologic evaluation, all but 2 skunks and the gray fox had brain lesions. The most consistently affected brain region was the frontal lobe (16 animals of 16 tested, 100%), followed by the cerebral cortex (46/53, 87%), brainstem (22/29, 76%), thalamus (20/29, 69%), hippocampus (17/29, 59%), and cerebellum (12/33, 36%). Brain lesions had a multifocal and random distribution, affected gray and white matter, and sometimes exhibited a periventricular distribution. Lesions primarily consisted of regions of malacia and inflammation (Figure 3, panel A). Acidophilic neuronal necrosis was prominent and often associated with satellitosis (Figure 3, panel B) or karyorrhectic debris (Figure 3, panel C). Laminar neuronal necrosis within the hippocampus was striking in some cases (Figure 3, panel D). Low-to-moderate numbers of predominantly lymphocytes and plasma cells infiltrated the leptomeninges and Virchow-Robin spaces, often extending into the parenchyma (Figure 3, panels A, B). In some presumptively subacute lesions, reactive histiocytes predominated. Histopathologic lesions in the brain were absent to mild in all skunks. In the 2 opossum joeys, brain lesions primarily affected the cerebral meninges.

**Table 1:** Results for avian influenza virus results for wild mammals by specimen type, grouped by species, United States, 2022*

Animal	Test	No. positive or present/no. tested or examined (%)								
		Nasal swab	OP swab	Nasal and OP swab	Tracheal swab	Brain	Heart	Lung	Liver	Lymphoid system
Red fox	PCR	20/22 (90)	15/18 (83)	9/9 (100)	5/7 (71)	31/32 (96)	7/8 (87)	12/13 (92)	NA	NA
	Lesions	NA	NA	NA	NA	38/38 (100)	25/37 (68)	35/38 (92)	13/32 (41)	18/32 (56)
	IHC	NA	NA	NA	NA	13/17 (76)	3/5 (60)	2/15 (13)	1/3 (33)	0/3 (0)
Skunk	PCR	NA	1/1 (100)	NA	NA	3/3 (100)	NA	3/3 (100)	2/2 (100)†	2/2 (100)†
	Lesions	NA	NA	NA	NA	3/5 (60)	0/5 (0)	5/5 (100)	5/5 (100)	5/5 (100)
	IHC	NA	NA	NA	NA	NA	NA	2/2 (100)	5/5 (100)	1/1 (100)
Raccoon	PCR	1/1 (100)	3/3 (100)	NA	NA	1/1 (100)	NA	NA	NA	NA
	Lesions	NA	NA	NA	NA	4/4 (100)	1/4 (25)	3/4 (75)	1/4 (25)	2/4 (50)
	IHC	NA	NA	NA	NA	1/1 (100)	1/1 (100)	1/1 (100)	NA	1/1 (100)
Opossum	PCR	NA	2/2 (100)	2/2 (100)	NA	2/2 (100)	NA	NA	NA	NA
	Lesions	NA	NA	NA	NA	1/2 (50)	0/2 (0)	0/2 (0)	1/2 (50)	0/2 (0)
	IHC	NA	NA	NA	NA	2/2 (100)	NA	1/2 (50)	NA	NA
Bobcat	PCR	2/2 (100)	2/2 (100)	NA	NA	2/2 (100)	1/2 (50)	2/2 (100)	NA	NA
	Lesions	NA	NA	NA	NA	2/2 (100)	2/2 (100)	1/2 (50)	1/2 (50)	2/2 (100)
	IHC	NA	NA	NA	NA	1/2 (50)	NA	0/2 (0)	NA	NA
Coyote	PCR	NA	NA	1/1 (100)	1/1 (100)	NA	NA	NA	NA	NA
	Lesions	NA	NA	NA	NA	1/1 (100)	1/1 (100)	1/1 (100)	A	0/1 (0)
	IHC	NA	NA	NA	NA	NA	NA	NA	NA	NA
Gray fox	PCR	NA	NA	1/1 (100)	0/1 (0)	0/1 (0)	NA	NA	NA	NA
	Lesions	NA	NA	NA	NA	0/1 (0)	0/1 (0)	0/1 (0)	0/1 (0)	0/1 (0)
	IHC	NA	NA	NA	NA	NA	NA	NA	NA	NA
Fisher	PCR	NA	1/1 (100)	NA	NA	1/1 (100)	NA	1/1 (100)	1/1 (100)	1/1 (100)
	Lesions	NA	NA	NA	NA	1/1 (100)	0/1 (0)	1/1 (100)	1/1 (100)	1/1 (100)
	IHC	NA	NA	NA	NA	NA	NA	NA	NA	NA
Total PCR results		23/25 (92)	24/27 (88)	13/13 (100)	6/9 (66)	40/42 (95)	8/10 (80)	18/19 (94)	3/3 (100)†	3/3 (100)†
Total lesion results		NA	NA	NA	NA	50/54 (93)	29/53 (55)	46/54 (85)	22/47 (47)	28/48 (58)
Total IHC results		NA	NA	NA	NA	17/22 (77)	4/6 (67)	6/22 (27)	6/8 (78)	2/5 (40)

*Lesions were detected by using histopathologic analysis. A, major autolysis; IHC, immunohistochemical analysis; NA, not assessed; OP, oropharyngeal. †Sample includes pooled liver and spleen.

Multifocal necrotizing interstitial pneumonia was the second most commonly reported microscopic lesion (n = 47). Alveoli contained fibrin, edema, hemorrhage, macrophages, and occasionally neutrophils (Figure 3, panel E). Alveolar septa were thickened by neutrophils, macrophages, perivascular lymphocytes and plasma cells, and aggregates of fibrin. Type II pneumocyte hyperplasia was rare, and few animals had bronchopneumonia. Most lung lesions were acute, except in 1 fox and 1 bobcat. Concurrent lungworm parasitism was noted in 3 raccoons, 1 bobcat, and the fisher.

Multifocal myocardial necrosis, most commonly affecting the left ventricular wall, was present in roughly half of the animals (n = 29). Mineralization of affected cardiomyocytes was often noted (Figure 3, panel F). Mild lymphoplasmacytic or histiocytic infiltrates and regions of fibrosis were infrequently reported in conjunction with myocardial necrosis. No heart lesions were observed in the striped skunks, the opossum joeys, the fisher, or the gray fox.

Random foci of acute liquefactive to coagulative hepatic necrosis were observed in 22 animals and in all 5 striped skunks. Dystrophic mineralization of hepatocytes and mixed inflammation associated with regions of necrosis were reported in some cases.

Lymphoid depletion was observed in 28 animals and was most consistent in the striped skunks and raccoons. Lymphoid necrosis was prominent in the spleen (n = 5), lymph nodes (n = 4), and Peyer’s patches of the ileum (n = 1) of the striped skunks and in the thymus and intestinal Peyer’s patches of 1 raccoon.

Foci of inflammation or necrosis were rarely observed in the kidney, tongue, and gastrointestinal tract in the red fox kits. A focal area of acute pancreatic necrosis and multifocal lymphoplasmacytic pancreatitis were noted in 1 red fox each. With the exception of a few small clusters of lymphocytes and plasma cells within the photoreceptor layer of the retina of 1 red fox, no lesions were reported in any of the examined eyes from other red foxes, including the 3 red foxes reported as clinically blind. The 1 gray fox had no microscopic lesions.

We performed IHC analysis for avian influenza antigen on a variety of tissues in 29 animals (Table 1). Of the 13 red foxes with immunoreactivity in the brain, positively staining neurons were detected in the cerebral cortex (n = 13), thalamus (n = 6), hippocampus (n = 3), and brainstem (n = 1) (Figure 3, panel G).

Immunoreactivity within lung tissue was limited to scattered mild staining of epithelial cells and interstitial macrophages (Figure 3, panel H) in 2 of 15 red foxes, both striped skunks and the 1 raccoon. One of 2 opossums had strong immunoreactivity within pneumocytes and to a lesser extent alveolar macrophages and bronchial and bronchiolar epithelium. Neither bobcat had immunoreactivity in the lung.

Three of 5 red foxes and the 1 raccoon that had IHC analysis performed on heart showed immunoreactivity of scattered cardiac myofibers and interstitial macrophages surrounding foci of necrosis. Immunoreactivity within hepatocytes surrounding necrotic foci was present in all 5 striped skunks, whereas only 1 of 3 red foxes tested had immunoreactivity in the liver.

A variety of sample types from 64 animals were tested by rRT-PCR in National Animal Health Laboratory Network laboratories using several assays that can detect IAV (Table 1; Appendix Table 5). With 1 exception (assay performed on formalin-fixed, paraffin-embedded brain tissue), all animals with nonnegative rRT-PCR results had >1 confirmed positive at NVSL. Most samples were uniformly positive using the general IAV, H5 subtype, and H5 clade 2.3.4.4b subtype rRT-PCRs, when performed (Appendix Table 5). Of 35 animals with >2 sample types tested, brain samples frequently had the strongest amplification signal (n = 21). Pooled nasal and oropharyngeal swab specimens had greater detection rates than did nasal or oropharyngeal swab specimens.

**Figure 4:**
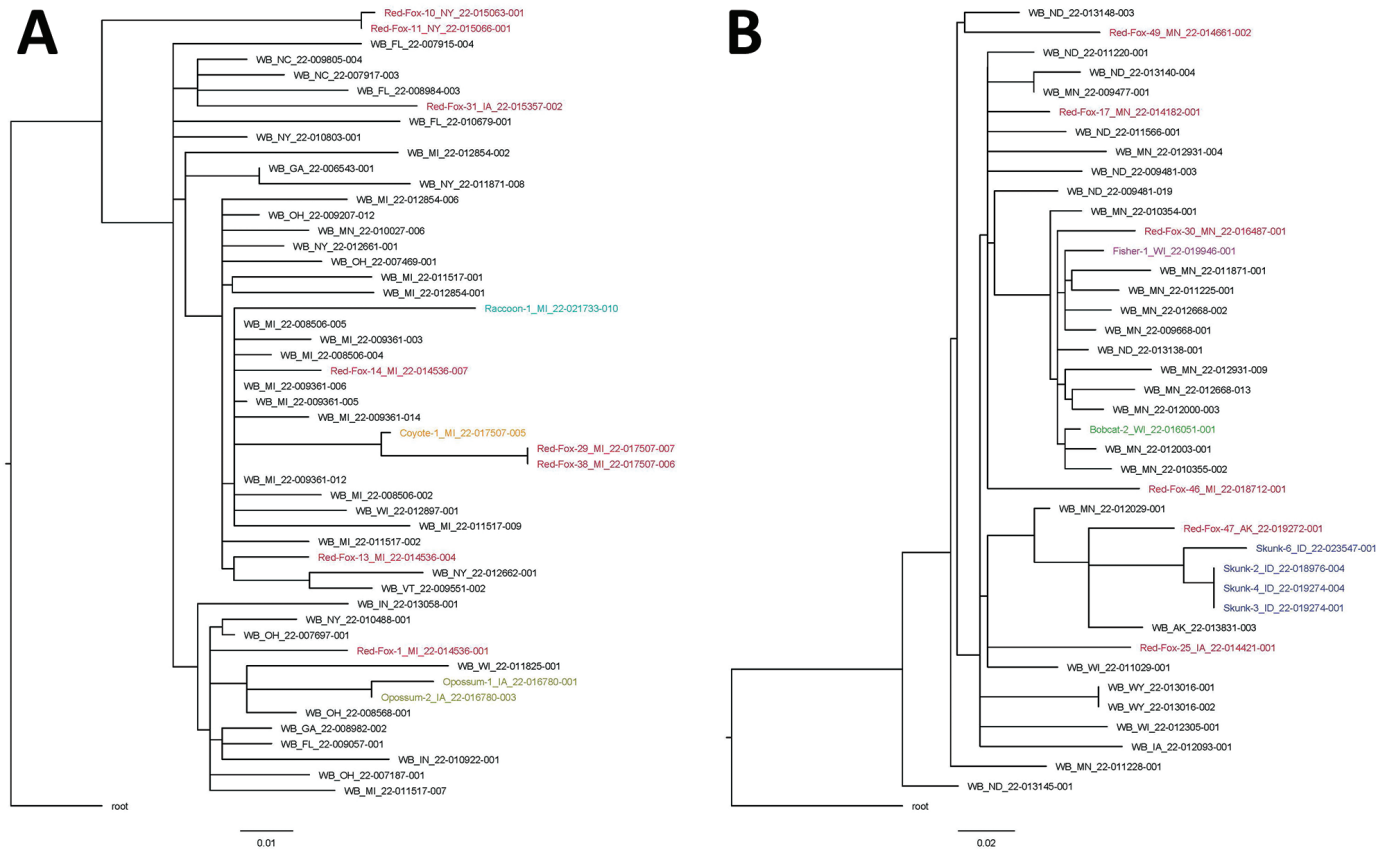
Single-nucleotide polymorphism phylogenetic trees for highly pathogenic avian influenza A(H5N1) clade 2.3.4.4b virus in mammals and wild birds. A) Genotype B1.2. Data from red fox are shown in red, raccoon in teal, coyote in orange, and Virginia opossum in gold. B) Genotype B3.2. Data from red fox are shown in red, fisher in purple, bobcat in green, and skunk in blue. Trees are rooted to the reference sequence A/Fancy_Ck/NL/FAV33/2021. WB, wild bird. Scale bars indicate nucleotide substitutions per site.

Full genome sequence data were obtained directly from 77 samples representing 48 animals across 7 species from 10 states. Sequences were deposited in GISAID (https://gisaid.org) (Appendix Table 6). Among the 48 animals, 9 different H5N1 genotypes were identified according to the specific combinations of Eurasian and North American gene segments (Table 2). All but 1 of these genotypes have at least North American polymerase basic 1 and nucleoprotein genes and represent reassortments of the Newfoundland-like H5N1 2.3.4.4b virus initially introduced into the Americas by the Atlantic Flyway with North American wild bird–origin influenza viruses (*2**,**3*). The 1 unreassorted virus from a red fox in Alaska represents a separate introduction of the H5N1 2.3.4.4b into the Americas based on phylogenetic analysis and high sequence identity to Asian-origin H5N1 viruses across all 8 segments (a Japan-like virus likely introduced via the Bering Strait) (*13*). The PB2 E627K substitution previously associated with mammalian adaptation (*14*) was identified at the consensus level in three animals as well as at the subconsensus level (mixed population) in 3 additional animals (Table 2). The PB2 D701N mutation was identified at the consensus level in 1 animal and at the subconsensus level in 3 animals (Table 2). The PB2 T271A mutation was not detected at consensus or subconsensus levels in any animal. Detections of PB2 E627K and D701N mutations are diverse in their geographic locations and genotypes. Analysis of all sequences indicated infections resulted from regional spillovers from wild birds, as shown by a subset of genotypes B1.2 and B3.2 (Appendix Figure) and their phylogenetic trees (Figure 4).

**Table 2:** Genotype analysis for complete sequences of H5N1 highly pathogenic avian influenza virus from mammal species, including substitutions in the PB2 gene at the consensus and subconsensus level, grouped by species, United States, 2022*

Animal	A3	B1.1	B1.2	B2	B3.1	B3.2	B4	Minor 02	Minor 15	Total
Red fox	1	7	6 E627K (1), D701N† (1)	8 E627K† (2), D701N† (1)	4 D701N (1)	6 E627K (1)	1	2	2	37
Skunk						4				4
Raccoon			1 E627K† (1)	1 E627K (1)						2
Opossum		2								2
Bobcat						1				1
Coyote			1 D701N† (1)							1
Fisher						1				1
Total	1	9	8	9	4	12	1	2	2	48

*Values are no. detections of PB2 substitutions. PB2, polymerase basic protein 2. †Substitutions were detected at the PB2 subconsensus level (mixed); those without a dagger were detected at the consensus level.

Ancillary testing for other viruses was performed on many of these animals and concurrent viral infections were identified in 4 animals (Appendix Table 7). One red fox had concurrent *Escherichia coli* enteritis and septicemia. No anticoagulant rodenticides were detected in a liver sample from the gray fox.

## Discussion

This case series highlights multiple detections of HPAI virus Eurasian lineage H5 clade 2.3.4.4b in wild terrestrial mammals in the United States. Our findings build on previous reports of natural infections with HPAI virus in red foxes from the Netherlands in 2021 (*15*) and add to the global list of species susceptible to H5N1 HPAI virus (*16*).

Red foxes are the most represented species in this report. Intrinsic factors related to exposure and infection risk could explain this finding, including opportunistic dietary preferences, likelihood of sharing environments with infected birds, abundance of immunologically naive animals present during the onset of the avian outbreak, and potentially increased susceptibility to infection in this species. Many of the red foxes were found in urban or periurban environments, and extrinsic factors, such as human interest in the highly visible animals, might have led to increased public reporting. Raccoons, skunks, opossums, and coyotes are also generalist mesopredators frequently encountered in urban and periurban areas (*17*), and reasons why these species were less represented are unclear. Although serologic evidence of exposure to AIVs has been documented in many wild mammal species, few experiments have investigated the susceptibility of wild mammals to these viruses, and even fewer specifically to HPAI H5N1 virus (*18*). Additional studies on the susceptibility of mammal species to infection with the currently circulating strains of HPAI H5N1 virus might be warranted, especially in light of the unprecedented reassortment of the Newfoundland-like virus with North American wild bird origin influenza viruses (*3*).

Neurologic signs were the primary clinical observation in this report, consistent with reports of HPAI H5N1 infections in mammals in Europe (*7*,*15*) and in infected birds of prey (*19*,*20*). Although widespread lesions and viral detection from multiple tissues are consistent with systemic infections in those mammals, necrotizing and nonsuppurative meningoencephalitis and acute interstitial pneumonia were the primary microscopic lesions, followed by myocardial necrosis, hepatic necrosis, and lymphoid depletion. Those findings are also consistent with lesion distribution in previous reports of natural and experimental HPAI virus infections in mammals (*7*,*15*,*21*,*22*) and raptors (*19*,*20*,*23*).

Brain and heart lesions were absent or mild in the striped skunks in this study; hepatic necrosis, lymphoid necrosis, and interstitial pneumonia were the predominant findings, corresponding to the distribution of virus as detected by IHC analysis and rRT-PCR. A similar distribution of lesions has been reported in domestic cats naturally infected with HPAI H5N1 virus (*24*). Therefore, the typical constellation of lesions associated with HPAI virus infection cannot be assumed to be uniform across mammalian families.

Consistent with the brain being the most frequently and strongly positive sample for rRT-PCR detection in most species in this study, 17 of 22 brains tested by IHC analysis had detectable immunoreactivity. Conversely, immunoreactivity within the lungs was rare and scattered in most species, despite the severity of gross and microscopic lung lesions. The cause of this discrepancy is unclear but might relate to early pulmonary clearance of the virus or cytokine-induced pulmonary injury, which has been reported in experimental infection trials in laboratory mammals (*25*).

rRT-PCR was the most sensitive method for IAV detection in this study, and there were only 4 cases in which HPAI virus was detected by rRT-PCR but tissues lacked immunoreactivity. In 2 red foxes that were either weakly positive by rRT-PCR or positive on nasal swab specimen only, lack of tissue immunoreactivity was probably caused by absence or limited amounts of HPAI virus in tissues. Detecting HPAI virus in these 2 foxes might have been clinically incidental because both foxes had concurrent infections that could have caused additional illness. Similarly, the 1 gray fox had no microscopic lesions and was weakly positive on pooled nasal and oropharyngeal swab specimens only, and this detection could have represented early or subclinical infection or simply mucosal carriage of HPAI virus. In our study, oropharyngeal swab specimens were generally more sensitive for the detection of HPAI virus in foxes than were nasal or intestinal/rectal swab specimens, consistent with higher oropharyngeal viral shedding reported in experimental infections of red foxes (*21*).

Most clinically ill mammals in this study died or were euthanized because of disease progression, similar to outcomes reported in other natural infections with HPAI H5N1 virus in mammals (*7*,*15*,*24*,*26*,*27*). However, clinical resolution of neurologic signs was documented in 1 juvenile red fox.

Adult red foxes, striped skunks, coyotes, and Virginia opossums infected with HPAI virus were not identified in this study. Reperant et al. did not observe clinical signs in 6- to 10-month-old foxes experimentally infected with HPAI virus (*21*). Reasons that clinical infections have been primarily restricted to young mammals are unknown but could include naive immune systems in juveniles, different exposure risks across age groups, and behavioral differences that might make ill adult mammals less likely to be encountered. Both bobcats were co-infected with parvovirus, which may have contributed to the development of clinical disease caused by HPAI virus infection in these adults.

Ingestion of birds infected with HPAI virus is presumed to be the most likely source of infection in wild mammals, and 3 red foxes in this study had evidence of bird ingestion. Wild birds, including waterfowl, are a typical or occasional component of the natural diet in those species, and infection following ingestion of HPAI virus‒positive birds has been confirmed in multiple mammals (*21*,*26**–**28*) and in raptors and scavenging birds (*19*,*20*,*23*). Clear evidence of mammal-to-mammal transmission was not apparent in these cases. However, horizontal transmission of HPAI H5N1 virus has been documented in experimentally infected domestic cats (*27*) and ferrets (*29*), and transmission from an infected parent or conspecific cannot be ruled out as a potential source of infection in some of the mammals in this study, particularly within affected littermates.

The scattered geographic and temporal distribution of the HPAI virus‒infected mammals in this study suggests that these infections represent sporadic spillover events into individual animals sharing the landscape with HPAI virus‒infected wild birds. This theory is supported by sequencing data from mammalian samples, confirming the presence of several different genotypes that have also been documented emerging and circulating in the United States in wild birds (*3*). In mammals, sustained deaths caused by HPAI H5N1 virus have thus far only been reported in seals (*30*), and methods of transmission responsible for the outbreak remain unclear. The PB2 gene E627K substitution that has been associated with mammal adaptation (*14*) and another PB2 mutation of concern D701N were identified in 10 (21%) of 48 mammals in this study, and a similar rate of mutations has been detected in HPAI virus‒infected wild mammals in Canada (*31*). Continued vigilance is warranted as ongoing spillover of avian influenza viruses into mammalian hosts could potentially result in further reassortment or adaptation of these viruses to broader host ranges (*32*,*33*).

In summary, we demonstrate that multiple North America wild terrestrial mammal species are susceptible to natural infection with HPAI H5N1 virus of Eurasian lineage goose/Guangdong H5 clade 2.3.4.4b, probably by ingestion of infected wild birds. Neurologic signs were the primary clinical manifestation, and HPAI virus infection warrants consideration as a differential diagnosis along with more common causes of neurologic disease in wild mammals. Given the ongoing nature of the HPAI virus outbreak and the detection of genetic substitutions concerning for mammalian adaptation, surveillance for HPAI virus in wild mammals would contribute to a better understanding of the distribution and evolution of these viruses in free-ranging wildlife.

Appendix Additional information on highly pathogenic avian influenza A(H5N1) virus clade 2.3.4.4b infections in wild terrestrial mammals, United States, 2022.
