# Supplementary material for: Highly Pathogenic Avian Influenza A(H5N1) Virus Clade 2.3.4.4b Infections in Wild Terrestrial Mammals, United States, 2022
Source: Emerg Infect Dis. 2023 Dec;29(12):2451–60. doi: 10.3201/eid2912.230464 (PMC10683806; doi:10.3201/eid2912.230464)
Supplement: Appendix — Additional information on highly pathogenic avian influenza A(H5N1) virus clade 2.3.4.4b infections in wild terrestrial mammals, United States, 2022. [file 23-0464-Techapp-s1.pdf]

Article DOI: https://doi.org/10.3201/eid2912.230464

EID cannot ensure accessibility for supplementary materials supplied by authors. Readers who have difficulty accessing supplementary content should contact the authors for assistance.

Highly Pathogenic Avian Influenza A(H5N1) Virus Clade 2.3.4.4b Infections in Wild Terrestrial Mammals, United States, 2022

Appendix

Appendix Table 1. Reference sequences used for single nucleotide polymorphism analysis.

Segment	Reference used	GenBank accession	GISAID ID
PB2	A/Blue-Winged Teal/Alberta/39/2020(H4N6)	MZ171362.1	NA
PB1	EPI2049863|A/chicken/NL/FAV-0033/2021	NA	EPI_ISL_12968823
PA	EPI2049864|A/chicken/NL/FAV-0033/2021	NA	EPI_ISL_12968823
HA	EPI2049865|A/chicken/NL/FAV-0033/2021	NA	EPI_ISL_12968823
NP	A/mallard/Nevada/AH0190913/2021(H11N6)	OL462886.1	NA
NA	EPI2049867|A/chicken/NL/FAV-0033/2021	NA	EPI_ISL_12968823
MP	EPI2049868|A/chicken/NL/FAV-0033/2021	NA	EPI_ISL_12968823
NS	EPI2049869|A/chicken/NL/FAV-0033/2021	NA	EPI_ISL_12968823

NA = not applicable.

Appendix Table 2. Summary of signalment, date found, days in human care (if applicable), state and county found, and clinical outcome of wild mammals naturally infected with highly pathogenic avian influenza, grouped by species and listed in order of date found.

Case ID		Species	Sex	Age class	Date found	Days in care	State	County	Outcome
Red fox	1	Vulpes vulpes	F	Juv	4/1/22	< 1	MI	Macomb	D
Red fox	2	Vulpes vulpes	M	Juv	4/1/22	<1	NY	Cayuga	E
Red fox	3	Vulpes vulpes	NR	NR	4/2/22	NA	AK	Aleutians west	D
Red fox	4*	Vulpes vulpes	NR	Juv	4/7/22	<1	NY	Erie	E
Red fox	5*	Vulpes vulpes	NR	Juv	4/7/22	>1	NY	Erie	E
Red fox	6*	Vulpes vulpes	F	Juv	4/9/22	>8	WI	Jefferson	A
Red fox	7*	Vulpes vulpes	F	Juv	4/9/22	2	WI	Jefferson	D
Red fox	8	Vulpes vulpes	M	Juv	4/9/22	>1	NY	Monroe	E
Red fox	9	Vulpes vulpes	F	Juv	4/11/22	<1	NY	Tompkins	E
Red fox	10*	Vulpes vulpes	F	Juv	4/11/22	<1	NY	St. Lawrence	E
Red fox	11*	Vulpes vulpes	M	Juv	4/11/22	>1	NY	St. Lawrence	E
Red fox	12	Vulpes vulpes	F	Juv	4/12/22	5	WI	Dane	E
Red fox	13	Vulpes vulpes	M	Juv	4/14/22	< 1	MI	Lapeer	D
Red fox	14	Vulpes vulpes	F	Juv	4/14/22	< 1	MI	St. Clair	D
Red fox	15	Vulpes vulpes	M	Juv	4/19/22	< 1	WI	Rock	E
Red fox	16	Vulpes vulpes	M	Juv	4/19/22	<1	NY	Erie	D
Red fox	17	Vulpes vulpes	NR	Juv	4/20/22	NA	MN	Anoka	D
Red fox	18	Vulpes vulpes	M	Juv	4/24/22	< 1	WI	Waushara	E
Red fox	19	Vulpes vulpes	F	Juv	4/24/22	< 1	WI	Dane	E
Red fox	20	Vulpes vulpes	M	Juv	4/25/22	<1	MN	Dakota	E
Red fox	21	Vulpes vulpes	F	Juv	4/25/22	<1	MN	Hennepin	E
Red fox	22*	Vulpes vulpes	M	Juv	5/3/22	1	WI	Adams	E
Red fox	23*	Vulpes vulpes	F	Juv	5/3/22	1	WI	Adams	E
Red fox	24	Vulpes vulpes	F	Juv	5/4/22	>8	WI	Dane	A
Red fox	25	Vulpes vulpes	M	Juv	5/4/22	NA	IA	Hancock	E
Red fox	26	Vulpes vulpes	M	Juv	5/4/22	<1	NY	Niagara	E
Red fox	27*	Vulpes vulpes	F	Juv	5/5/22	1	WI	Grant	E

Case ID		Species	Sex	Age class	Date found	Days in care	State	County	Outcome
Red fox	28*	Vulpes vulpes	M	Juv	5/5/22	1	WI	Grant	D
Red fox	29	Vulpes vulpes	F	Juv	5/5/22	NA	MI	Osceola	E
Red fox	30	Vulpes vulpes	NR	Juv	5/8/22	<1	MN	Otter Tail	E
Red fox	31	Vulpes vulpes	F	Juv	5/9/22	2	IA	Cedar	E
Red fox	32	Vulpes vulpes	M	Juv	5/10/22	NA	MI	Midland	D
Red fox	33	Vulpes vulpes	M	Juv	5/10/22	NA	MI	Midland	D
Red fox	34	Vulpes vulpes	F	Juv	5/10/22	NA	MN	Stearns	D
Red fox	35	Vulpes vulpes	M	Juv	5/11/22	<1	NY	Allegany	E
Red fox	36	Vulpes vulpes	M	Juv	5/14/22	<1	MI	Gladwin	D
Red fox	37	Vulpes vulpes	M	Juv	5/15/22	<1	MI	Gladwin	D
Red fox	38	Vulpes vulpes	M	Juv	5/18/22	NA	MI	Ostego	D
Red fox	39	Vulpes vulpes	F	Juv	5/18/22	<1	MI	Bay	D
Red fox	40	Vulpes vulpes	M	Juv	5/19/22	NA	MI	Osceola	E
Red fox	41*	Vulpes vulpes	F	Juv	5/23/22	NA	ND	Dickey	E
Red fox	42*	Vulpes vulpes	M	Juv	5/23/22	NA	ND	Dickey	E
Red fox	43	Vulpes vulpes	M	Juv	5/24/22	NA	UT	Salt Lake	E
Red fox	44	Vulpes vulpes	M	Juv	5/26/22	NA	UT	Salt Lake	E
Red fox	45	Vulpes vulpes	M	Juv	5/31/22	8	MI	Mackinac	E
Red fox	46	Vulpes vulpes	M	Juv	6/1/22	<1	MI	Muskegon	D
Red fox	47	Vulpes vulpes	M	Juv	6/6/22	NA	AK	Nome	E
Red fox	48	Vulpes vulpes	NR	Juv	NR	<1	MN	NR	E
Red fox	49	Vulpes vulpes	F	Juv	NR	<1	MN	NR	E
Red fox	50	Vulpes vulpes	M	Juv	NR	<1	MN	NR	E
Skunk	1*	Mephitis mephitis	F	Juv	6/6/22	>3	ID	Latah	D
Skunk	2*	Mephitis mephitis	M	Juv	6/6/22	>3	ID	Latah	E
Skunk	3*	Mephitis mephitis	F	Juv	6/7/22	>3	ID	Latah	E
Skunk	4*	Mephitis mephitis	F	Juv	6/7/22	>3	ID	Latah	E
Skunk	5*	Mephitis mephitis	M	Juv	6/7/22	>3	ID	Latah	E
Skunk	6	Mephitis mephitis	NR	Juv	7/21/22	NA	ID	Ada	D
Raccoon	1	Procyon lotor	F	Ad	5/11/22	NA	MI	Iron	E
Raccoon	2	Procyon lotor	NR	Juv	6/8/22	NA	WA	Franklin	D
Raccoon	3	Procyon lotor	NR	Juv	6/8/22	NA	WA	Franklin	D
Raccoon	4	Procyon lotor	F	Juv	6/30/22	NA	WA	Stevens	NR
Bobcat	1	Lynx rufus	F	Ad	4/24/22	NA	WI	Lincoln	E
Bobcat	2	Lynx rufus	F	Ad	5/6/22	NA	WI	Lincoln	E
Opossum	1*	Didelphis virginiana	M	Juv	5/18/22	1	IA	Benton	E
Opossum	2*	Didelphis virginiana	F	Juv	5/18/22	2	IA	Benton	D
Coyote	1	Canis latrans	F	Juv	5/3/22	NA	MI	Wexford	E
Fisher	1	Pekania pennanti	M	Ad	4/14/22	NA	WI	St. Croix	E
Gray fox	1	Urocyon cinereoargenteus	F	Ad	6/2/22	NA	MI	Clinton	D

* = indicates animals that were known or strongly suspected to be littermates. NR = not recorded. F = female. M = male. Ad = adult. Juv = juvenile. NA = not applicable. A = alive at time of publication. D = died naturally. E = euthanized.

Appendix Table 3 Summary of reported clinical signs demonstrated by wild mammals naturally infected with highly pathogenic avian influenza virus, grouped by species.

Case ID		Neurologic signs, any reported	Seizures	Ataxia	Tremors	Lack of fear	Hyper-salivation	Lethargy	Febrile	Other, reported in ≤5 animals
Red fox	1	X	X	X					X	
Red fox	2	X	X							
Red fox	4	X	X				X	X	X	unconscious
Red fox	5	X	X				X	X	X	unconscious
Red fox	6	X		X				X		
Red fox	7	X		X				X		vocalization
Red fox	8	X					X			blindness, circling
Red fox	9	X	X		X			X		vocalization
Red fox	10	X	X							vocalization
Red fox	11	X	X							diarrhea, vocalization
Red fox	12							X		
Red fox	13	X	X	X					X	
Red fox	14	X	X	X					X	
Red fox	15	X	X		X					vocalization
Red fox	16		X		X		X	X		

Red fox	19	X		X					diarrhea, dyspnea
Red fox	20	X	X	X	X			X	
Red fox	21	X			X			X	
Red fox	22	X	X		X			X	
Red fox	23	X			X			X	torticollis
Red fox	24								dehydrated, thin
Red fox	25	X		X		X			
Red fox	26		X				X	X	
Red fox	27	X	X		X			X	nystagmus, torticollis
Red fox	28	X	X						
Red fox	29	X							
Red fox	30	X	X						blindness, circling
Red fox	31	X	X						blindness, grimace
Red fox	35	X	X						dull, vomiting
Red fox	36	X							
Red fox	37	X		X				X	recumbent
Red fox	39	X		X				X	
Red fox	40	X	X	X					
Red fox	41	X		X					unconscious
Red fox	42	X		X					paralysis
Red fox	43	X	X						
Red fox	44	X		X				X	
Red fox	45	X							
Red fox	46	X				X		X	
Red fox	47	X				X			circling
Red fox	48	X	X	X	X			X	
Red fox	49	X	X	X	X			X	
Red fox	50	X		X				X	
Skunk	1	X		X	X			X	dyspnea
Skunk	2	X		X	X			X	dyspnea
Skunk	3	X	X		X			X	
Skunk	4	X	X		X			X	
Skunk	5	X	X		X			X	
Raccoon	1	X							
Raccoon	4	X		X					
Bobcat	1	X			X	X			
Bobcat	2	X				X		X	dyspnea
Opossum	1	X	X						
Opossum	2	X	X						
Coyote	1	X		X				X	
Fisher	1	X		X		X		X	
Gray fox	1	X		X		X			circling

X = reported. Blank = not reported.

Appendix Table 4. Histopathologic lesions in wild mammals naturally infected with highly pathogenic avian influenza virus infection.

		Brain lesions: Necrotizing and inflammatory meningoencephalitis									
Case ID		Brainstem	Cerebellum	Hippocampus	Thalamus	Cortex	Frontal lobe	Myocardial necrosis	Interstitial pneumonia	Lymphoid depletion	Hepatic necrosis
Red fox	1					+		+	+	+	-
Red fox	2	+	+	+		+		-	+	-	-
Red fox	7	+	+	+	+	+		+	+	+	+
Red fox	8	-	-	-	+	-	+	+	-	-	-
Red fox	9	+	+	+	+	+	+	+	+	-	-
Red fox	10					+		-	+	-	-
Red fox	12	-	-	-	-	-	+	+	+	+	+
Red fox	13					+		+	+	+	-
Red fox	14					+		+	+	+	-
Red fox	15							+	+	+	+
Red fox	17	+	+	+	+	+			+		
Red fox	18	+	+	+	+	+	+	+	+	+	-
Red fox	19	+			+	+	+	+	+	+	+
Red fox	20	+	-	-	+	+		+	+		+
Red fox	21	+	-	+	+	+		+	+	-	+
Red fox	22	+	+	-	+	+	+	+	+	-	+
Red fox	23	+	-	+	+	+	+	+	+	-	+
Red fox	25					+		+	+	+	+
Red fox	26	+		+	+	+	+	+	+	-	+
Red fox	27	+	+		+	+	+	+	+	-	-
Red fox	28	+	+			+	+	-	+	+	-
Red fox	29					+		+	+	+	+
Red fox	30		-		-	+					
Red fox	31		-			+		+	+	+	+
Red fox	32					+		-	+	A	A
Red fox	33					+		-	+	A	A
Red fox	34	-	-	+	+	+		+	+	-	-
Red fox	35	+	-		+	+	+	-	+	-	-
Red fox	36					+		-	+	-	-
Red fox	37					+		-	+	A	A
Red fox	38					+		-	-	-	A
Red fox	39					+		-	+	A	A
Red fox	49	+	+	+		+		+	+	-	-
Red fox	41		-	+		+	+	-	+	+	+
Red fox	42		-	+		+	+	+	+	+	-
Red fox	43	+	+	+	+	+		+	+	+	-
Red fox	44			-	+	+		+	-	+	-
Red fox	45					+		-	+	+	-
Red fox	46					+		+	+	+	-
Skunk	1	+	-	-	-	-		-	+	+	+
Skunk	2	+	-	-	-	-		-	+	+	+
Skunk	3	-	-	-	-	+		-	+	+	+
Skunk	4	-	-	-	-	-		-	+	+	+
Skunk	5	-	-	-	-	-		-	+	+	+
Raccoon	1					+		-	+	-	-

Case ID		Brain lesions: Necrotizing and inflammatory meningoencephalitis									
		Brainstem	Cerebellum	Hippocampus	Thalamus	Cortex	Frontal lobe	Myocardial necrosis	Interstitial pneumonia	Lymphoid depletion	Hepatic necrosis
Raccoon	2	+	-	-	-	+		-	-	+	-
Raccoon	3	+	+	-	-	+		-	+	+	+
Raccoon	4	-	-	+	+	+		+	+	-	-
Bobcat	1	+	-	+	+	+	+	+	+	+	-
Bobcat	2	+	-	+	+	+	+	+	-	+	+
Opossum	1					+		-	-	-	+
Opossum	2		-			-		-	-	-	-
Coyote	1					+		+	+	-	A
Fisher	1		-	+	+	+	+	-	+	+	+
Gray fox	1					-		-	-	-	-

Blank = not evaluated. - = lesion not present. + = lesion present. A = too autolyzed to interpret.

Appendix Table 5. Cycle threshold (CT) results for quantitative real time PCR for influenza A virus (IAV), IAV H5 subtype, and avian influenza virus H5 2.3.4.4b subtype assays in wild mammals, by tissue or sample type.

Case ID	Assay	Nasal swab	OP swab	Nasal & OP swab	Tracheal swab	Brain	Lung	Heart	Intestine*	Liver	Kidney	Spleen
Red fox 1	IAV	n/d	n/d	19.4	31.6	13.9	n/d	n/d	n/d	n/d	n/d	n/d
	H5	n/d	n/d	17.5	28.8	12.4	n/d	n/d	n/d	n/d	n/d	n/d
	2.3.4.4	n/d	n/d	21.5	32	14.7	n/d	n/d	n/d	n/d	n/d	n/d
Red fox 2	IAV	n/d	n/d	n/d	n/d	15	n/d	n/d	n/d	n/d	n/d	n/d
Red fox 3	IAV	28.4	n/d	n/d	n/d	n/d	n/d	n/d	n/d	n/d	n/d	n/d
	H5	24.5	n/d	n/d	n/d	n/d	n/d	n/d	n/d	n/d	n/d	n/d
	2.3.4.4	28.2	n/d	n/d	n/d	n/d	n/d	n/d	n/d	n/d	n/d	n/d
Red fox 4	IAV	n/d	n/d	n/d	n/d	17.2	n/d	n/d	n/d	n/d	n/d	n/d
	H5	n/d	n/d	n/d	n/d	8.6	n/d	n/d	n/d	n/d	n/d	n/d
	2.3.4.4	n/d	n/d	n/d	n/d	13.1	n/d	n/d	n/d	n/d	n/d	n/d
Red fox 5	IAV	n/d	n/d	n/d	n/d	12.6	n/d	n/d	n/d	n/d	n/d	n/d
	H5	n/d	n/d	n/d	n/d	12.9	n/d	n/d	n/d	n/d	n/d	n/d
	2.3.4.4	n/d	n/d	n/d	n/d	17.2	n/d	n/d	n/d	n/d	n/d	n/d
Red fox 6	IAV	38.8	n/d	n/d	n/d	n/d	n/d	n/d	n/d	n/d	n/d	n/d
	H5	36	n/d	n/d	n/d	n/d	n/d	n/d	n/d	n/d	n/d	n/d
	2.3.4.4	38.6	n/d	n/d	n/d	n/d	n/d	n/d	n/d	n/d	n/d	n/d
Red fox 7	IAV	38.9	28.5	28.3	32.1	37.8	31.3	n/d	n/d	n/d	n/d	n/d
	H5	N	33.6	26.2	29.6	N	29	n/d	n/d	n/d	n/d	n/d
	2.3.4.4	37.8	36.8	29	31.8	39.6	31.7	n/d	n/d	n/d	n/d	n/d
Red fox 8	IAV	n/d	n/d	n/d	n/d	38.1 (FFPE)	n/d	n/d	n/d	n/d	n/d	n/d
Red fox 9	IAV	n/d	n/d	n/d	n/d	n/d	34.792	n/d	n/d	n/d	n/d	n/d
	2.3.4.4	n/d	n/d	n/d	n/d	n/d	34.6	n/d	n/d	n/d	n/d	n/d
Red fox 10	IAV	n/d	n/d	n/d	n/d	17.104	n/d	n/d	n/d	n/d	n/d	n/d
	H5	n/d	n/d	n/d	n/d	15.4	n/d	n/d	n/d	n/d	n/d	n/d
	2.3.4.4	n/d	n/d	n/d	n/d	20	n/d	n/d	n/d	n/d	n/d	n/d
Red fox 11	IAV	n/d	n/d	n/d	n/d	27.093	n/d	n/d	n/d	n/d	n/d	n/d
	H5	n/d	n/d	n/d	n/d	22	n/d	n/d	n/d	n/d	n/d	n/d
	2.3.4.4	n/d	n/d	n/d	n/d	25.9	n/d	n/d	n/d	n/d	n/d	n/d
Red fox 12	IAV	N	35.5	n/d	n/d	N	38.6	40.1	N	n/d	n/d	n/d
	H5	n/d	34	n/d	n/d	37.4	N	N	N	n/d	n/d	n/d
	2.3.4.4	n/d	36	n/d	n/d	n/d	39.4	N	N	n/d	n/d	n/d
Red fox 13	IAV	n/d	n/d	36.3	37.4	15.6	n/d	n/d	N	n/d	n/d	n/d
	H5	n/d	n/d	34	34.2	15.5	n/d	n/d	n/d	n/d	n/d	n/d
	2.3.4.4	n/d	n/d	38.5	37.3	17.7	n/d	n/d	n/d	n/d	n/d	n/d
Red fox 14	IAV	n/d	n/d	28.6	28.6	11.9	n/d	n/d	N	n/d	n/d	n/d
	H5	n/d	n/d	25.9	26.3	10.4	n/d	n/d	n/d	n/d	n/d	n/d
	2.3.4.4	n/d	n/d	28.6	28.8	12.9	n/d	n/d	n/d	n/d	n/d	n/d
Red fox 15	IAV	n/d	n/d	n/d	32.9	17.6	24.4	34.6	37.5	n/d	n/d	n/d
	H5	n/d	n/d	n/d	28.2	13.9	21.3	26.2	34.8	n/d	n/d	n/d
	2.3.4.4	n/d	n/d	n/d	32.1	16.7	24.5	28.3	36	n/d	n/d	n/d
Red fox 16	IAV	n/d	n/d	n/d	n/d	15	n/d	n/d	n/d	n/d	n/d	n/d
	N1	n/d	n/d	n/d	n/d	14.4	n/d	n/d	n/d	n/d	n/d	n/d
	2.3.4.4	n/d	n/d	n/d	n/d	16.2	n/d	n/d	n/d	n/d	n/d	n/d
Red fox 17	IAV	n/d	n/d	n/d	n/d	13.4	n/d	n/d	n/d	n/d	n/d	n/d
	H5	n/d	n/d	n/d	n/d	14	n/d	n/d	n/d	n/d	n/d	n/d
	2.3.4.4	n/d	n/d	n/d	n/d	13.7	n/d	n/d	n/d	n/d	n/d	n/d
Red fox 18	IAV	36.9	29.1	n/d	n/d	29	24.5	35.4	37.5	n/d	n/d	n/d
	H5	N	24.8	n/d	n/d	26.6	20.9	34	37.5	n/d	n/d	n/d
	2.3.4.4	38.3	28.4	n/d	n/d	29.8	23.8	36	36	n/d	n/d	n/d
Red fox 19	IAV	34.6	N	n/d	n/d	N	N	N	N	n/d	n/d	n/d
	H5	32.9	n/d	n/d	n/d	n/d	N	N	N	n/d	n/d	n/d
	2.3.4.4	36	n/d	n/d	n/d	n/d	N	N	N	n/d	n/d	n/d
Red fox 20	IAV	n/d	n/d	n/d	n/d	15.9	n/d	n/d	n/d	n/d	n/d	n/d
	H5	n/d	n/d	n/d	n/d	16	n/d	n/d	n/d	n/d	n/d	n/d
	2.3.4.4	n/d	n/d	n/d	n/d	19	n/d	n/d	n/d	n/d	n/d	n/d
Red fox 21	IAV	n/d	n/d	n/d	n/d	22.4	n/d	n/d	n/d	n/d	n/d	n/d
	H5	n/d	n/d	n/d	n/d	22.1	n/d	n/d	n/d	n/d	n/d	n/d
	2.3.4.4	n/d	n/d	n/d	n/d	24.7	n/d	n/d	n/d	n/d	n/d	n/d
Red fox 22	IAV	29.8	23.8	n/d	n/d	13.5	32.3	33.3	34.5	n/d	n/d	n/d
	H5	29.1	22.2	n/d	n/d	14.6	30.7	33.7	33.8	n/d	n/d	n/d
	2.3.4.4	28.7	24.9	n/d	n/d	15	30.9	33.2	33.5	n/d	n/d	n/d
Red fox 23	IAV	39.8	32.9	n/d	n/d	16.4	34	33.5	N	n/d	n/d	n/d
	H5	N	30.7	n/d	n/d	16.5	32.1	32.8	38.1	n/d	n/d	n/d

Case ID	Assay	Nasal swab	OP swab	Nasal & OP swab	Tracheal swab	Brain	Lung	Heart	Intestine*	Liver	Kidney	Spleen
	2.3.4.4	36	33.6	n/d	n/d	16.4	32.5	32.4	37.9	n/d	n/d	n/d
Red fox 24	IAV	N	36	n/d	n/d	n/d	n/d	n/d	n/d	n/d	n/d	n/d
	H5	N	36.9	n/d	n/d	n/d	n/d	n/d	n/d	n/d	n/d	n/d
	2.3.4.4	N	35.9	n/d	n/d	n/d	n/d	n/d	n/d	n/d	n/d	n/d
Red fox 25	IAV	n/d	25.2	26.9	n/d	23.4	n/d	n/d	N	n/d	n/d	n/d
	H5	n/d	27.2	29	n/d	26	n/d	n/d	N	n/d	n/d	n/d
	2.3.4.4	n/d	27.4	28.5	n/d	N	n/d	n/d	n/d	n/d	n/d	n/d
Red fox 26	IAV	n/d	n/d	n/d	n/d	n/d	32.3	n/d	n/d	n/d	n/d	n/d
	H5	n/d	n/d	n/d	n/d	n/d	32	n/d	n/d	n/d	n/d	n/d
	2.3.4.4	n/d	n/d	n/d	n/d	n/d	33.3	n/d	n/d	n/d	n/d	n/d
Red fox 27	IAV	25.8	32.2	n/d	n/d	12.9	24.8	24.7	30.9	n/d	n/d	n/d
	H5	24.2	30.6	n/d	n/d	11.6	24.3	22.9	31.4	n/d	n/d	n/d
	2.3.4.4	27.6	35.1	n/d	n/d	14.8	28.2	28	34.3	n/d	n/d	n/d
Red fox 28	IAV	21.3	23.2	n/d	n/d	12.9	21.1	21.3	30.3	n/d	n/d	n/d
	H5	18.7	21.5	n/d	n/d	11.1	19.9	19	29.7	n/d	n/d	n/d
	2.3.4.4	22.8	25.7	n/d	n/d	14.7	23.7	23.6	33.5	n/d	n/d	n/d
Red fox 29	IAV	29.8	n/d	n/d	n/d	n/d	n/d	n/d	n/d	n/d	n/d	n/d
	H5	32.3	n/d	n/d	n/d	n/d	n/d	n/d	n/d	n/d	n/d	n/d
	2.3.4.4	34.1	n/d	n/d	n/d	n/d	n/d	n/d	n/d	n/d	n/d	n/d
Red fox 30	IAV	n/d	n/d	n/d	n/d	19.8	n/d	n/d	n/d	n/d	n/d	n/d
	H5	n/d	n/d	n/d	n/d	19.8	n/d	n/d	n/d	n/d	n/d	n/d
	2.3.4.4	n/d	n/d	n/d	n/d	19.9	n/d	n/d	n/d	n/d	n/d	n/d
Red fox 31	IAV	n/d	N	34.9	n/d	29.8	n/d	n/d	N	n/d	n/d	n/d
	H5	n/d	n/d	38.3	n/d	33	n/d	n/d	n/d	n/d	n/d	n/d
	2.3.4.4	n/d	n/d	N	n/d	30.9	n/d	n/d	n/d	n/d	n/d	n/d
Red fox 32	IAV	26.4	n/d	n/d	n/d	n/d	n/d	n/d	n/d	n/d	n/d	n/d
	H5	28.2	n/d	n/d	n/d	n/d	n/d	n/d	n/d	n/d	n/d	n/d
	2.3.4.4	29	n/d	n/d	n/d	n/d	n/d	n/d	n/d	n/d	n/d	n/d
Red fox 33	IAV	32.9	n/d	n/d	n/d	n/d	n/d	n/d	n/d	n/d	n/d	n/d
	H5	35.1	n/d	n/d	n/d	n/d	n/d	n/d	n/d	n/d	n/d	n/d
	2.3.4.4	36.3	n/d	n/d	n/d	n/d	n/d	n/d	n/d	n/d	n/d	n/d
Red fox 34	IAV	n/d	N	n/d	n/d	31.5	n/d	n/d	n/d	n/d	n/d	n/d
	H5	n/d	n/d	n/d	n/d	29.1	n/d	n/d	n/d	n/d	n/d	n/d
	2.3.4.4	n/d	n/d	n/d	n/d	30.5	n/d	n/d	n/d	n/d	n/d	n/d
Red fox 35	IAV	n/d	n/d	n/d	n/d	n/d	39.2	n/d	n/d	n/d	n/d	n/d
	H5	n/d	n/d	n/d	n/d	n/d	39.4	n/d	n/d	n/d	n/d	n/d
	2.3.4.4	n/d	n/d	n/d	n/d	n/d	N	n/d	n/d	n/d	n/d	n/d
Red fox 36	IAV	29.2	23.6	n/d	n/d	14.3	n/d	n/d	n/d	n/d	n/d	n/d
	H5	26.4	26.1	n/d	n/d	15.7	n/d	n/d	n/d	n/d	n/d	n/d
	2.3.4.4	30	26	n/d	n/d	16.8	n/d	n/d	n/d	n/d	n/d	n/d
Red fox 37	IAV	39.6	22.7	n/d	n/d	n/d	n/d	n/d	n/d	n/d	n/d	n/d
	H5	37.6	25.4	n/d	n/d	n/d	n/d	n/d	n/d	n/d	n/d	n/d
	2.3.4.4	38.9	25.1	n/d	n/d	n/d	n/d	n/d	n/d	n/d	n/d	n/d
Red fox 38	IAV	27	n/d	n/d	n/d	n/d	n/d	n/d	n/d	n/d	n/d	n/d
	H5	27	n/d	n/d	n/d	n/d	n/d	n/d	n/d	n/d	n/d	n/d
	2.3.4.4	27.3	n/d	n/d	n/d	n/d	n/d	n/d	n/d	n/d	n/d	n/d
Red fox 39	IAV	33.3	25	n/d	n/d	18.1	n/d	n/d	n/d	n/d	n/d	n/d
	H5	30.1	24.8	n/d	n/d	18.4	n/d	n/d	n/d	n/d	n/d	n/d
	2.3.4.4	32.6	34.9	n/d	n/d	19.6	n/d	n/d	n/d	n/d	n/d	n/d
Red fox 40	IAV	36.3	n/d	n/d	n/d	n/d	n/d	n/d	n/d	n/d	n/d	n/d
	H5	36.8	n/d	n/d	n/d	n/d	n/d	n/d	n/d	n/d	n/d	n/d
	2.3.4.4	37	n/d	n/d	n/d	n/d	n/d	n/d	n/d	n/d	n/d	n/d
Red fox 41	IAV	n/d	27.93	n/d	n/d	n/d	n/d	n/d	n/d	n/d	n/d	n/d
	H5	n/d	31.4	n/d	n/d	n/d	n/d	n/d	n/d	n/d	n/d	n/d
	2.3.4.4	n/d	30.57	n/d	n/d	n/d	n/d	n/d	n/d	n/d	n/d	n/d
Red fox 42	IAV	n/d	27.61	n/d	n/d	n/d	n/d	n/d	n/d	n/d	n/d	n/d
	H5	n/d	30.28	n/d	n/d	n/d	n/d	n/d	n/d	n/d	n/d	n/d
	2.3.4.4	n/d	30.55	n/d	n/d	n/d	n/d	n/d	n/d	n/d	n/d	n/d
Red fox 43	IAV	n/d	n/d	n/d	N	27.5	n/d	n/d	n/d	n/d	n/d	n/d
	H5	n/d	n/d	n/d	N	28	n/d	n/d	n/d	n/d	n/d	n/d
	2.3.4.4	n/d	n/d	n/d	n/d	29	n/d	n/d	n/d	n/d	n/d	n/d
Red fox 44	IAV	n/d	n/d	n/d	N	29.9	n/d	n/d	n/d	n/d	n/d	n/d
	H5	n/d	n/d	n/d	n/d	N	n/d	n/d	n/d	n/d	n/d	n/d
	2.3.4.4	n/d	n/d	n/d	n/d	33.8	n/d	n/d	n/d	n/d	n/d	n/d
Red fox 45	IAV	32.7	n/d	n/d	n/d	38.7	n/d	n/d	n/d	n/d	n/d	n/d
	H5	34.4	n/d	n/d	n/d	N	n/d	n/d	n/d	n/d	n/d	n/d
	2.3.4.4	39.5	n/d	n/d	n/d	39	n/d	n/d	n/d	n/d	n/d	n/d

Case ID	Assay	Nasal swab	OP swab	Nasal & OP swab	Tracheal swab	Brain	Lung	Heart	Intestine*	Liver	Kidney	Spleen
Red fox 46	IAV	24.9	26.4	n/d	n/d	20.5	n/d	n/d	n/d	n/d	n/d	n/d
	H5	21.7	27.6	n/d	n/d	22.6	n/d	n/d	n/d	n/d	n/d	n/d
	2.3.4.4	26.4	28.4	n/d	n/d	24.1	n/d	n/d	n/d	n/d	n/d	n/d
Red fox 47	IAV	32.9	n/d	n/d	n/d	n/d	26.4	n/d	33.4	n/d	n/d	n/d
	H5	30.4	n/d	n/d	n/d	n/d	24.7	n/d	32.0	n/d	n/d	n/d
	2.3.4.4	31.2	n/d	n/d	n/d	n/d	26.2	n/d	33.0	n/d	n/d	n/d
Red fox 48	IAV	n/d	n/d	24.3	n/d	n/d	n/d	n/d	n/d	n/d	n/d	n/d
	H5	n/d	n/d	24.1	n/d	n/d	n/d	n/d	n/d	n/d	n/d	n/d
	2.3.4.4	n/d	n/d	27.2	n/d	n/d	n/d	n/d	n/d	n/d	n/d	n/d
Red fox 49	IAV	n/d	n/d	33.7	n/d	n/d	n/d	n/d	n/d	n/d	n/d	n/d
	H5	n/d	n/d	32.1	n/d	n/d	n/d	n/d	n/d	n/d	n/d	n/d
	2.3.4.4	n/d	n/d	34.5	n/d	n/d	n/d	n/d	n/d	n/d	n/d	n/d
Red fox 50	IAV	n/d	n/d	23.9	n/d	n/d	n/d	n/d	n/d	n/d	n/d	n/d
	2.3.4.4	n/d	n/d	23.9	n/d	n/d	n/d	n/d	n/d	n/d	n/d	n/d
Skunk 2	IAV	n/d	n/d	n/d	n/d	14.5	20.5	n/d	n/d	n/d	n/d	n/d
	H5	n/d	n/d	n/d	n/d	11.1	19	n/d	n/d	n/d	n/d	n/d
	2.3.4.4	n/d	n/d	n/d	n/d	11.3	18.5	n/d	n/d	n/d	n/d	n/d
Skunk 3	IAV	n/d	n/d	n/d	n/d	n/d	19.1	n/d	n/d	19.4‡	n/d	n/d
	H5	n/d	n/d	n/d	n/d	n/d	18.5	n/d	n/d	19.6‡	n/d	n/d
	2.3.4.4	n/d	n/d	n/d	n/d	n/d	18.2	n/d	n/d	20.1‡	n/d	n/d
Skunk 4	IAV	n/d	n/d	n/d	n/d	22	19.2	n/d	n/d	20.4‡	n/d	n/d
	H5	n/d	n/d	n/d	n/d	20.7	19	n/d	n/d	20.5‡	n/d	n/d
	2.3.4.4	n/d	n/d	n/d	n/d	23.6	20	n/d	n/d	20.8‡	n/d	n/d
Skunk 6	IAV	n/d	28.7	n/d	n/d	20.9	n/d	n/d	n/d	n/d	n/d	n/d
	H5	n/d	N	n/d	n/d	N	n/d	n/d	n/d	n/d	n/d	n/d
	2.3.4.4	n/d	27.3	n/d	n/d	19.9	n/d	n/d	n/d	n/d	n/d	n/d
Raccoon 1	IAV	35.6	39.9	n/d	n/d	23.1	n/d	n/d	n/d	n/d	n/d	n/d
	H5	36.5	N	n/d	n/d	23.9	n/d	n/d	n/d	n/d	n/d	n/d
	2.3.4.4	34.3	39.6	n/d	n/d	25	n/d	n/d	n/d	n/d	n/d	n/d
Raccoon 2	IAV	n/d	32.1	n/d	n/d	n/d	n/d	n/d	n/d	n/d	n/d	n/d
	H5	n/d	29.2	n/d	n/d	n/d	n/d	n/d	n/d	n/d	n/d	n/d
	2.3.4.4	n/d	32.9	n/d	n/d	n/d	n/d	n/d	n/d	n/d	n/d	n/d
Raccoon 4	IAV	n/d	34.5	n/d	n/d	n/d	n/d	n/d	n/d	n/d	n/d	n/d
	H5	n/d	31.6	n/d	n/d	n/d	n/d	n/d	n/d	n/d	n/d	n/d
	2.3.4.4	n/d	34	n/d	n/d	n/d	n/d	n/d	n/d	n/d	n/d	n/d
Opossum 1	IAV	n/d	23.1	23.4	n/d	17.5	n/d	n/d	N	n/d	n/d	n/d
	H5	n/d	25	25.1	n/d	n/d	n/d	n/d	n/d	n/d	n/d	n/d
	2.3.4.4	n/d	24.8	24.7	n/d	24.9	n/d	n/d	n/d	n/d	n/d	n/d
Opossum 2	IAV	n/d	30.9	28.1	n/d	16.2	n/d	n/d	N	n/d	n/d	n/d
	H5	n/d	35	32.4	n/d	n/d	n/d	n/d	n/d	n/d	n/d	n/d
	2.3.4.4	n/d	36	33.2	n/d	23.9	n/d	n/d	n/d	n/d	n/d	n/d
Bobcat 1	IAV	34.3	35.2	n/d	n/d	20.4	27.6	38.8	34.7	n/d	n/d	n/d
	H5	29.8	30.5	n/d	n/d	20.5	28.5	N	36	n/d	n/d	n/d
	2.3.4.4	33.9	35	n/d	n/d	19.1	27.2	39.1	34	n/d	n/d	n/d
Bobcat 2	IAV	33.1	31.9	n/d	n/d	30.3	N	N	n/d	n/d	n/d	n/d
	H5	28.7	27.3	n/d	n/d	31.3	39.6	N	34.9	n/d	n/d	n/d
	2.3.4.4	32.8	31.6	n/d	n/d	29.9	N	N	n/d	n/d	n/d	n/d
Coyote 1	IAV	n/d	n/d	28	29.3	n/d	n/d	n/d	n/d	n/d	n/d	n/d
	H5	n/d	n/d	29	27.6	n/d	n/d	n/d	n/d	n/d	n/d	n/d
	2.3.4.4	n/d	n/d	29.6	30.9	n/d	n/d	n/d	n/d	n/d	n/d	n/d
Gray fox 1	IAV	n/d	n/d	37.7	N	N	n/d	n/d	n/d	n/d	n/d	n/d
	H5	n/d	n/d	37.4	N	N	n/d	n/d	n/d	n/d	n/d	n/d
	2.3.4.4	n/d	n/d	35.7	N	N	n/d	n/d	n/d	n/d	n/d	n/d
Fisher 1	IAV	n/d	29.7	n/d	n/d	17	22.2	n/d	33.9	33	28.7	31
	H5	n/d	28.6	n/d	n/d	19.4	24.5	n/d	34.8	34.9	30.5	32.8
	2.3.4.4	n/d	28.6	n/d	n/d	19.3	24.8	n/d	35.4	34.4	30.2	31.5

OP = oropharyngeal; * = includes rectal swabs, intestinal swabs, and intestine sample types; N = not detected; n/d = not done; ‡ = pooled liver and spleen.

Appendix Table 6 GISAID IDs for sequences from wild mammals with avian influenza virus infections.

Manuscript ID	Text ID	GISAID ID
Red fox 1	22-014536-001	EPI_ISL_16240634
Red fox 1	22-014536-002	EPI_ISL_16240635

Manuscript ID	Text ID	GISAID ID
Red fox 3	22-016300-001	EPI_ISL_17260669
Red fox 4	22-015064-001	EPI_ISL_16243264
Red fox 5	22-015065-001	EPI_ISL_16243273
Red fox 7	22-013774-002	EPI_ISL_13052717
Red fox 10	22-015063-001	EPI_ISL_16243253
Red fox 11	22-015066-001	EPI_ISL_17260668
Red fox 13	22-014536-004	EPI_ISL_15078245
Red fox 14	22-014536-007	EPI_ISL_16240636
Red fox 14	22-014536-008	EPI_ISL_16240637
Red fox 15	22-013774-013	EPI_ISL_17260662
Red fox 15	22-013774-014	EPI_ISL_13052719
Red fox 15	22-014746-007	EPI_ISL_17260664
Red fox 17	22-014182-001	EPI_ISL_13052720
Red fox 18	22-013774-008	EPI_ISL_17260661
Red fox 18	22-013774-009	EPI_ISL_13052718
Red fox 20	22-014660-001	EPI_ISL_13052722
Red fox 21	22-014660-002	EPI_ISL_13052723
Red fox 22	22-014746-008	EPI_ISL_15078246
Red fox 22	22-014746-010	EPI_ISL_13052725
Red fox 22	22-014746-015	EPI_ISL_17260665
Red fox 22	22-014746-016	EPI_ISL_13052726
Red fox 25	22-014421-001	EPI_ISL_13052721
Red fox 27	22-014746-021	EPI_ISL_17260666
Red fox 27	22-014746-023	EPI_ISL_13052727
Red fox 28	22-014746-026	EPI_ISL_17260667
Red fox 28	22-014746-029	EPI_ISL_13052728
Red fox 28	22-014746-030	EPI_ISL_15078247
Red fox 29	22-017507-007	EPI_ISL_17260677
Red fox 30	22-016487-001	EPI_ISL_15078249
Red fox 31	22-015357-002	EPI_ISL_16243970
Red fox 32	22-017507-008	EPI_ISL_17260678
Red fox 33	22-017056-001	EPI_ISL_17260672
Red fox 36	22-021733-007	EPI_ISL_17260687
Red fox 36	22-018712-005	EPI_ISL_17260679
Red fox 37	22-021733-003	EPI_ISL_17260685
Red fox 38	22-017507-006	EPI_ISL_17260676
Red fox 39	22-018712-003	EPI_ISL_18090482
Red fox 39	22-021733-005	EPI_ISL_17260686
Red fox 41	22-017061-001	EPI_ISL_17260673
Red fox 42	22-017061-002	EPI_ISL_17260674
Red fox 43	22-016402-001	EPI_ISL_17260670
Red fox 46	22-018712-001	EPI_ISL_15078253
Red fox 46	22-021733-002	EPI_ISL_17260684
Red fox 47	22-019272-001	EPI_ISL_17260681
Red fox 48	22-014661-001	EPI_ISL_13052724
Red fox 49	22-014661-002	EPI_ISL_17260663
Skunk 2	22-018976-004	EPI_ISL_17260680
Skunk 3	22-019274-001	EPI_ISL_15078254
Skunk 4	22-019274-004	EPI_ISL_17260682
Skunk 8	22-023547-001	EPI_ISL_17260689
Raccoon 1	22-021733-010	EPI_ISL_17260688
Raccoon 2	22-018406-002	EPI_ISL_15078252
Opossum 1	22-016780-001	EPI_ISL_15078250
Opossum 2	22-016780-003	EPI_ISL_17260671
Bobcat 2	22-016051-001	EPI_ISL_15078248
Coyote 1	22-017507-005	EPI_ISL_17260675
Fisher 1	22-019946-001	EPI_ISL_17260683

Appendix Table 7. Summary of results for select viral etiologies in wild mammals naturally infected with highly pathogenic avian influenza. All testing performed is by rRT-PCR, unless otherwise indicated.

Case ID	HPAI	AIV†	RV*	CAV	CDV	CHV	CPV/ FPV	CCoV	CRCoV	CPIV	FHV	FCV	FCoV	SARS-CoV-2
Red fox 1	P		N		N†									
Red fox 2	P		N		N†									
Red fox 3	P													
Red fox 4	P													

Case ID		HPAI	AIV†	RV*	CAV	CDV	CHV	CPV/ FPV	CCoV	CRCoV	CPIV	FHV	FCV	FCoV	SARS-CoV-2
Red fox	5	P													
Red fox	6	P				N									
Red fox	7	P	P	N	N	N	N	N	N	N	N				
Red fox	8	P		N		N†									
Red fox	9	P	P	N											
Red fox	10	P		N											
Red fox	11	P		N		N									
Red fox	12	P	N	N	N	N	N	N	N	N	N				
Red fox	13	P		N		N†									
Red fox	14	P		N		N†									
Red fox	15	P	N	N	N	N	N	N	N	N	N				
Red fox	16	P													
Red fox	17	P	P	N		N†									
Red fox	18	P	P		N	N	N	N	N	N	N				
Red fox	19	P	N	N	P	N	N	N	N	N	N				
Red fox	20	P	P	N		N†									
Red fox	21	P	P	N		N†									
Red fox	22	P	P	N	N	N	N	N	N	N	N				
Red fox	23	P	P	N	N	N	N	N	N	N	N				
Red fox	24	P													
Red fox	25	P	P	N		N									
Red fox	26	P		N											
Red fox	27	P	P		N	N	N	N	N	N	N				
Red fox	28	P	P		N	N	N	N	N	N	N				
Red fox	29	P	P												
Red fox	30	P		N		N†									
Red fox	31	P	P	N		N									
Red fox	32	P													
Red fox	33	P													
Red fox	34	P	P	N		N†									
Red fox	35	P		N											
Red fox	36	P		N											
Red fox	37	P		N											
Red fox	38	P													
Red fox	39	P		N											
Red fox	40	P													
Red fox	41	P				N									
Red fox	42	P				N									
Red fox	43	P		N											
Red fox	44	P		N											
Red fox	45	P		N											
Red fox	46	P		N											
Red fox	47	P		P		N									
Red fox	48	P													
Red fox	49	P													
Red fox	50	P													
Skunk	1		P		N	N									
Skunk	2	P	P												
Skunk	3	P	P												
Skunk	4	P	P												
Skunk	5		P												
Skunk	6	P		N(PCR)		N^									
Raccoon	1	P		N											
Raccoon	2	P	P												
Raccoon	3		P												
Raccoon	4	P	P												
Bobcat	1	P	N			N		P				N	N	N	N
Bobcat	2	P	P			N		P				N	N	N	N
Opossum	1	P	P	N											
Opossum	2	P	P	N											
Coyote	1	P													
Fisher	1	P				N		N							N
Gray fox	1	P		N											

HPAI = highly pathogenic avian influenza virus. AIV = avian influenza virus. RV = rabies virus. CAV = canine adenovirus. CDV = canine distemper virus. CHV = canine herpesvirus. CPV/FPL = canine parvovirus/feline panleukopenia virus. CCoV = canine coronavirus type 1. CRCoV = canine respiratory coronavirus. CPIV = canine parainfluenza virus. FHV = feline herpesvirus. FCV = feline calicivirus. FCOV = feline coronavirus. SARS-CoV-2 = severe acute respiratory syndrome coronavirus 2 (COVID-19). P = positive result. N = negative result. Blank = not performed. * = fluorescent antibody testing. † = immunohistochemical assay.

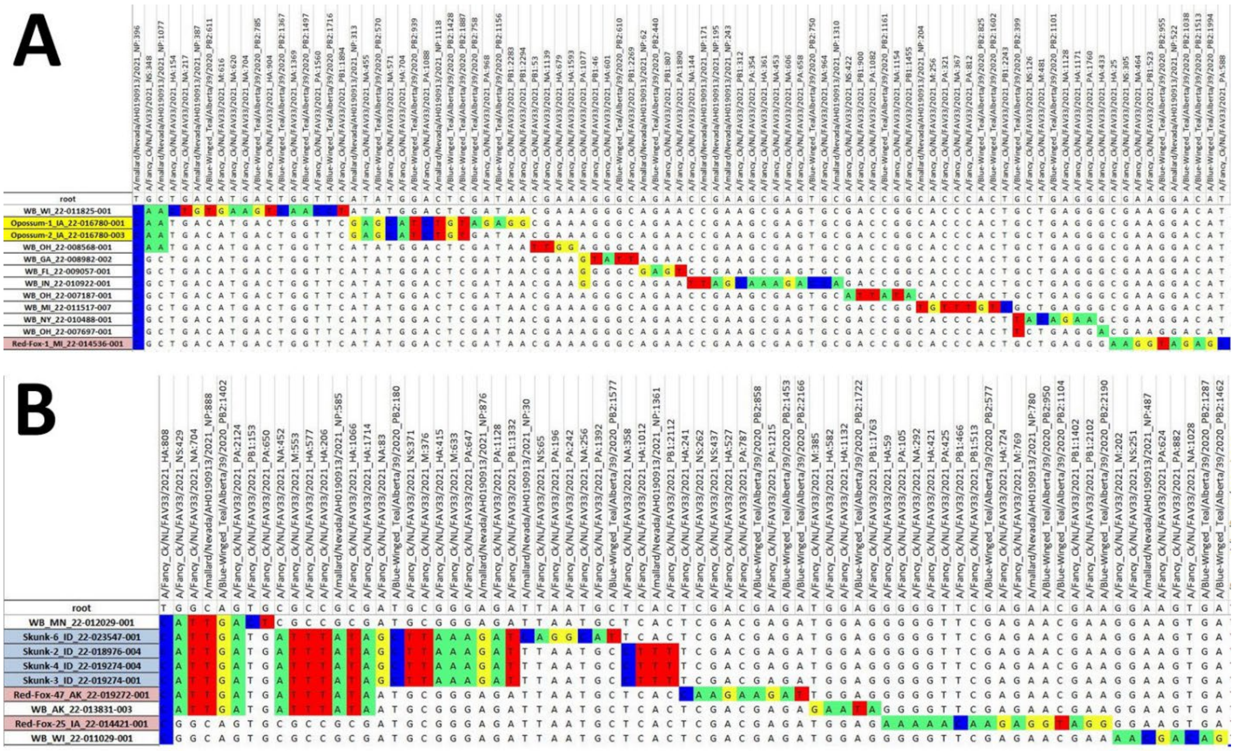

Appendix Figure. A) Example of a subset of B1.2 virus single-nucleotide polymorphism analysis using whole genome data for H5N1 clade 2.3.4.4b highly pathogenic avian influenza in mammals and wild birds. Data for 3 samples from 1 red fox and 2 samples from one Virginia opossum are highlighted in red and yellow, respectively. Each column represents the nucleotide position relative to the reference virus. B), Example of a subset of B3.2 virus single-nucleotide polymorphism analysis using whole genome data for H5N1 clade 2.3.4.4b highly pathogenic avian influenza in mammals and wild birds. Data from 4 samples from 4 skunks and 3 samples from 2 red foxes are highlighted in blue and red, respectively. Each column represents the nucleotide position relative to the reference virus.
